# Material values, environmental attitudes, and pro-environmental behaviors among future physicians in a coastal setting

**DOI:** 10.1038/s41598-026-47832-9

**Published:** 2026-04-23

**Authors:** Marium Mahmoud Abdulla, Abdullah Masoud Ghandy, Abdalla Hisham Ibrahim, Asmaa Mahmoud Abdulaziz, Bahiga Hafez Daoud

**Affiliations:** 1 Community Medicine and Public Health Department, Faculty of Medicine, Alexandria University, Azarita, Champollion Street, Alexandria, 21521 Egypt; 2 Faculty of Medicine, Alexandria University, Azarita, Champollion Street, Alexandria, 21521 Egypt; 3 Industrial Medicine and Occupational Health, Community Medicine Department, Faculty of Medicine, Alexandria University, Azarita, Champollion Street, Alexandria, 21521 Egypt

**Keywords:** Material values, Materialism, Environmental attitudes, Pro-environmental behaviors, Medical students, Environmental sciences, Environmental social sciences, Environmental studies, Health care, Health humanities, Psychology, Psychology

## Abstract

Human–environment interactions are increasingly strained by unsustainable consumption patterns shaped by cultural values and social norms. Materialism, defined as prioritizing possessions and financial success, has been consistently linked to lower environmental concern and reduced engagement in pro-environmental behaviors (PEBs). However, PEBs are not uniform: private behaviors (e.g., personal conservation) and public behaviors (e.g., advocacy, group participation) may have distinct determinants, including social visibility and status considerations. This study examined material values, environmental attitudes, and both private and public PEBs among medical students in Alexandria, a coastal Mediterranean city, using a cross-sectional survey with quota sampling. Students reported moderate materialism, high environmental attitudes, moderate private PEBs, and low public PEBs. Bivariate analyses indicated negative associations between material values (MV) and environmental attitudes and private PEBs, while public PEBs were linked to sociodemographic factors and MV. Regression models revealed that private PEBs were predicted by nationality and MV-centrality, whereas public PEBs—including environmental group membership, donations to environmental groups, and attendance of conferences on environmental issues—were differentially associated with MV orientations and cultural background. These findings highlight domain-specific determinants of environmental behavior and underscore the importance of embedding sustainability education and experiential engagement in medical curricula, tailored to diverse socio-cultural and economic contexts, to foster environmentally responsible practices among future healthcare professionals.

**Supplementary Information:**

The online version contains supplementary material available at 10.1038/s41598-026-47832-9.

## Introduction

Human–environment interactions are increasingly strained by unsustainable consumption patterns that are deeply shaped by cultural values and social norms. Materialism, defined as prioritizing acquisition and possessions, acts as a powerful cultural driver of resource-intensive lifestyles and ecological degradation^1–3^.

A substantial body of research has demonstrated that material values (MV) are generally associated with less favorable environmental attitudes and lower engagement in pro-environmental behaviors (PEBs)^4–6^. A meta-analysis by Hurst et al. confirmed a robust negative relationship between materialism and both environmental attitudes and behaviors across diverse populations^7^. However, emerging research suggests that this relationship may be more nuanced, with some forms of public pro-environmental engagement potentially linked to status signaling or social visibility rather than intrinsic environmental concern^8^. Importantly, PEBs are not a homogeneous construct. Prior research distinguishes between private PEBs (e.g., personal consumption and conservation practices) and public PEBs (e.g., advocacy, group participation), which differ in underlying motivations, visibility, and social signaling functions^9^. Distinguishing between these domains is therefore critical for accurately understanding the pathways through which MV and environmental attitudes translate into behavior.

The value–attitude–behavior framework posits that broad value orientations shape specific attitudes, which in turn influence behavioral outcomes^10^. In this context, MV are expected to be associated with environmental attitudes, which in turn are linked to engagement in PEBs. At the individual level, MV and environmental attitudes represent key socio-psychological determinants of PEBs, a critical pathway through which individuals respond to environmental challenges. Environmental attitudes—encompassing beliefs, affective responses, and behavioral intentions toward the environment^11^, remain among the strongest predictors of PEBs^12,13^. Together, MV and environmental attitudes form part of a broader cultural–psychological system influencing environmentally relevant decision-making. Prior research has also highlighted tensions between materialistic and pro-environmental value orientations, often conceptualized as a value conflict shaping consumer and lifestyle choices^14^. Early work in this field similarly demonstrated the role of materialism in shaping environmentally relevant behaviors such as recycling and post-purchase actions^15,16^.

Despite this growing evidence base, most studies have focused on general populations in high-income settings^8,17,18^. Far less is known about these dynamics among future physicians—an occupational group whose role extends beyond clinical care to promoting environmental sustainability within healthcare systems. Given that healthcare contributes nearly 5% of global greenhouse gas emissions^19^, understanding the value orientations and behaviors of future physicians is increasingly relevant to planetary health and sustainable development^20^.

This study is conducted in Alexandria, a coastal Mediterranean city facing documented environmental pressures, including sea-level rise and resource constraints^21,22^. While the present study does not directly measure climate risk perception or adaptation behaviors, this context provides an environmental backdrop rather than an analytical variable for examining how socio-cultural factors relate to environmental attitudes and behaviors among medical students. Alexandria Faculty of Medicine draws students from diverse sociodemographic backgrounds across the Arab region, offering an opportunity to explore how these characteristics intersect with shared educational experiences.

To our knowledge, this is the first study to jointly examine MV, environmental attitudes, and both private and public PEBs among medical students in this population and regional context. The study aimed to: (1) assess levels of MV, environmental attitudes, and private and public PEBs; (2) identify sociodemographic correlates of these constructs; (3) examine their interrelationships; and (4) determine predictors of private and public PEBs. Accordingly, the study examineed the relationships between these constructs. This framing positions the study as an analysis of environmental attitudes and behaviors within a coastal context, without attributing explanatory power to climate vulnerability itself.

## Methods

This cross-sectional study employed non-probability quota sampling to proportionally represent fifth-year medical students at Alexandria Faculty of Medicine, Egypt. Fifth-year students were selected as they are nearing entry into professional practice, a stage at which value systems and attitudes are expected to be more fully developed. Quotas were constructed based on the distribution of students across nationality groups—Egyptian, from Gulf Cooperation Council (GCC) countries, or other Arab countries (OAC)—within the fifth-year cohort, as obtained from student affairs records, ensuring proportional representation within this defined sampling frame. Although this approach does not aim to represent the broader population of Egypt or the region, it was appropriate for examining associations within this specific academic cohort. Accordingly, the sampling strategy is best characterized as non-probability quota sampling within a defined population. A total of 405 students participated between 27 September and 31 December 2024. Recruitment occurred via online student groups, and consenting students completed an Arabic-language questionnaire administered through Google Forms. An English version of the study tool is provided as Supplementary file S1 online.

### Measures

1. Personal characteristics: Age, gender, nationality, and self-reported monthly pocket money in Egyptian pounds (EGP). Gender was recorded as male or female, consistent with societal and institutional norms in Egypt.
2. Material values: MV were assessed using the validated Arabic 15-item Material Values Scale^23^. This version was adapted by Alsoudi et al. from the original scale developed by Richins and Dawson^24^. In line with Alsoudi et al.^23^, one item in the 15-item version (I put less emphasis on material things than most peo-ple I know) was replaced with an equivalent item from the original 18-item scale^17^ (I usually buy only the things I need) to enhance cultural relevance. The original Arabic version had high internal consistency (Cronbach’s α of 0.82) among an Arabic population in Oman^23^. The scale comprises three subscales—success, centrality, and happiness—and includes six reverse-coded items. Cronbach’s α for MV scale in the present study was 0.730
3. Environmental attitudes: It was initially assessed using the validated Arabic version^25^, consisting of two subscales from Özden’s scale: attitudes toward environmental issues (5 items) and solutions (10 items), of which five items were reverse-coded^26^. Though the original version reported good Cronbach’s α^26^, and the Arabic version reported Cronbach’s α exceeding 0.80 for sub-scales and total scale in Jordan^25^, yet the present study found low internal consistency, with Cronbach’s α = 0.294. Items may reflect multiple underlying dimensions (e.g., environmental concern, responsibility, behavioral intentions, and views on development and policy). Item-level diagnostics guided by item–total correlations and Cronbach’s α were conducted, and poorly performing items were removed, yielding a revised 7-item scale (α = 0.723). The original instrument is available in Özden^26^; due to copyright restrictions, items are not reproduced here. An adapted version and the final 7-item scale are provided in Supplementary File S2.
4. Pro-environmental behaviors and their frequency: PEBs were adapted and translated from Tsai et al.^27^, covering six private and four public behaviors. Items were culturally modified (e.g., car driving adapted to driving/riding as most students don’t own a car; signing petitions was omitted due to the rarity of such activities in Egypt; protests adapted to environment issue-relatd conference attendance (e.g., on climate change), reflecting the local sociopolitical context in Egypt, in which environmental protests are not present). Internal consistency was acceptable for private PEBs (Cronbach’s α = 0.727) but lower for public PEBs (α = 0.600), reflecting the heterogeneous nature of the latter. Accordingly, public PEBs were not treated as a single latent construct; instead, each public behaviour was analysed separately. Private PEBs were retained as a composite scale and used in subsequent analyses.

Items for MV, attitudes, and private PEBs were rated on a 5-point Likert scale from 1 (strongly disagree/never) to 5 (strongly agree/always). Scale means were calculated, with higher scores indicating stronger materialism, more favorable environmental attitudes, or higher engagement in private PEBs. Scores were categorized as low (1.00–2.33), moderate (2.34–3.67), or high (3.68–5.00). Public PEBs were coded as binary responses (yes/no).

Ethical approval was granted by the Institutional Review Board of the Faculty of Medicine, Alexandria (IRB No: 00012098). The study followed the Declaration of Helsinki. Informed consent was obtained electronically, participation was voluntary, and no identifiable data were collected.

### Sample size and stratification

Based on Gheith^28^ (mean PEB score = 2.57 ± 1.05, n = 296), the required sample was calculated using the formula n = (Z^2^ × SD^2^) / MOE^2^ where: SD = Standard deviation, MOE = Margin of error^29^. The minimum required sample size was 270, which was inflated to 405 (× 1.5) to preserve statistical power and proportional stratification. Using official enrollment lists, students were stratified as Egyptian (55%, n = 225), from OAC; including Jordan, Palestine, Syria, Iraq, Sudan, Libya, and Yemen; 35%, n = 140), and from GCC countries (10%, n = 40). Separate Google Forms were created for each group, with responses capped once quotas were reached.

### Data analysis

As all questions were required, no missing data occurred. Inconsistent entries for pocket money (n = 28) were coded as missing and excluded only from analyses involving that variable. Data were analyzed using IBM SPSS Statistics, version 23.0. Descriptive statistics were presented as frequencies and percentages for categorical data or mean ± standard deviation (SD), 95% confidence interval (CI), median alongside interquartile range (IQR), for continuous data. Normality was tested with Shapiro–Wilk test, showing non-normal distributions. Accordingly, Mann–Whitney U, Kruskal–Wallis H, and Spearman’s tests were applied for primary analysis, with chi-square used for categorical comparisons. Parametric tests (independent t-test, one-way ANOVA, and Pearson’s correlation) were conducted as sensitivity analyses, yielded some differences in statistical significance compared with the primary non-parametric results, and are presented in Supplementary file S3 online. Multivariate analysis was conducted to control confounding bias, using linear regression for private PEBs and logistic regression for public PEBs, including all variables to avoid introducing omitted variable bias or suppression effects. A significance level of *p* < 0.05 was applied to all analyses.

## Results

### General characteristics of the studied population

A total of 405 fifth-year medical students participated in the study. The majority were Egyptian (55.6%, n = 225), followed by students from OAC (34.5%, n = 140) and the GCC countries (9.9%, n = 40). The mean age of participants was 22.5 years (SD ± 1.3), with a minimum of 21, and a maximum of 36 years. Most participants were male (64.7%). Reported monthly pocket money ranged widely, with a mean of 10,595 EGP and a median of 3500 EGP, reflecting a skewed distribution.

### Private pro-environmental behaviors

#### Material values, environmental attitudes, and Private Pro-Environmental Behaviors: levels and sociodemographic determinants (objectives 1 and 2)

Participants reported moderate levels of MV, and private PEBs. Within subscales, MV-success scores were low, while environmental attitudes were high; all other subscales showed moderate levels (Table 1). Significant associations were observed between nationality, gender, age, and pocket money and various dimensions, including MV-centrality and private PEBs (*p* < 0.05).

**Table 1:** Material values, environmental attitudes and private pro-environmental behaviors by student characteristics (n = 405).

	MV success	MV happiness	MV centrality	Total MV	Env. attitude	PEBs private
All participants (n = 405)						
Mean ± SD	2.25 ± 0.71	2.95 ± 0.75	2.93 ± 0.64	2.71 ± 0.54	4.40 ± 0.53	2.72 ± 0.84
[95% CI for mean]	[2.18–2.32]	[2.88–3.03]	[2.86–2.99]	[2.66–2.76]	[4.35–4.45]	[2.64–2.80]
Median (IQR)	2.20 (1.00)	3.00 (1.00)	3.00 (1.00)	2.73 (0.80)	4.57 (0.57)	2.67 (1.00)
Min–max	1.00–4.40	1.00–4.80	1.20–5.00	1.27–4.13	1.57–5.00	1.00–5.00
Mean ± SD [95% CI for Mean] Median (IQR)						
Nationality						
Egyptian (n = 225)	2.28 ± 0.71 [2.19–2.38] 2.20 (1.00)	2.99 ± 0.75 [2.89–3.09] 3.00 (1.20)	2.89 ± 0.60 [2.81–2.97] 3.00 (1.00)	2.72 ± 0.52 [2.65–2.79] 2.73 (0.77)	4.43 ± 0.49 [4.36–4.49] 4.57 (0.43)	2.59 ± 0.80 [2.48–2.69] 2.50 (1.17)
OAC (n = 140)	2.23 ± 0.72 [2.11–2.35] 2.20 (1.20)	3.00 ± 0.73 [2.88–3.12] 3.00 (1.00)	2.98 ± 0.71 [2.86–3.09] 3.00 (1.00)	2.74 ± 0.58 [2.64–2.83] 2.73 (0.83)	4.38 ± 0.57 [4.29–4.48] 4.42 (0.71)	2.92 ± 0.90 [2.77–3.07] 2.66 (1.00)
GCC (n = 40)	2.14 ± 0.62 [1.94–2.33] 2.10 (0.80)	2.60 ± 0.72 [2.37–2.83] 2.60 (1.00)	2.96 ± 0.63 [2.76–3.16] 3.00 (1.10)	2.57 ± 0.49 [2.41–2.72] 2.50 (0.58)	4.28 ± 0.63 [4.08–4.49] 4.42 (0.68)	2.77 ± 0.77 [2.52–3.04] 3.00 (1.29)
	*p* = 0.628	*p* = 0.015*	*p* = 0.686	*p* = 0.291	*p* = 0.374	*p* = 0.003*
Gender						
Male (n = 262)	2.27 ± 0.72 [2.18–2.36] 2.20 (1.00)	2.99 ± 0.73 [2.90–3.08] 3.00 (1.20)	2.84 ± 0.62 [2.77–2.92] 2.80 (0.80)	2.70 ± 0.53 [2.64–2.77] 2.66 (0.73)	4.36 ± 0.59 [4.28–4.43] 4.42 (0.61)	2.71 ± 0.88 [2.60–2.81] 2.66 (1.33)
Female (n = 143)	2.21 ± 0.68 [2.10–2.33] 2.20 (1.20)	2.88 ± 0.78 [2.75–3.01] 3.00 (1.20)	3.08 ± 0.65 [2.97–3.19] 3.20 (0.80)	2.72 ± 0.56 [2.63–2.82] 2.73 (0.73)	4.48 ± 0.42 [4.41–4.55] 4.57 (0.43)	2.74 ± 0.77 [2.62–2.87] 2.83 (1.00)
	*p* = 0.629	*p* = 0.167	*p* < 0.001*	*p* = 0.425	*p* = 0.199	*p* = 0.560
Correlation coefficient						
Age	r = − 0.123	r = − 0.009	r = − 0.111	r = − 0.096	r = 0.067	r = 0.126
	*p* = 0.012*	*p* = 0.856	*p* = 0.025*	*p* = 0.055	*p* = 0.178	*p* = 0.011*
Pocket money^$^	r = 0.038	r = − 0.005	r = 0.114	r = 0.063	r = − 0.092	r = 0.125
	*p* = 0.463	*p* = 0.916	*p* = 0.027*	*p* = 0.219	*p* = 0.075	*p* = 0.015*

MV: Material values; Env. attitude: Environmental Attitude; PEBs: Pro-environmental behaviors; SD: Standard deviation; CI: Confidence interval; IQR: Interquartile range; OAC: Other Arab countries; GCC: Gulf Cooperation Council. $: n = 377. Non-parametric tests (Mann–Whitney U and Kruskal–Wallis) were used for group comparisons, and Spearman’s correlation was used for correlations.

*Denotes statistically significant difference at 0.05 level.

#### Interrelationships between Material values, environmental attitudes, and private pro-environmental behaviors (objective 3)

Significant interrelationships were observed between materialism, environmental attitudes, and private PEBs (Table 2) (*p* < 0.05). Environmental attitude and private PEBs were negatively correlated with total MV and MV-success and were positively correlated with each other.

**Table 2:** Correlations among material values, environmental attitudes and private pro-environmental behaviors.

	MV success	MV happiness	MV centrality	Total MV	Env. attitude	PEBs private
MV success	–	–	–	–	–	–
MV happiness	–	–	–	–	–	–
MV centrality	–	–	–	–	–	–
Total MV	–	–	–	–	–	–
Total attitude	r = − 0.193 *p* = < 0.001*	r = − 0.085 *p* = 0.089	r = − 0.078 *p* = 0.115	r = − 0.147 *p* = 0.003*	–	–
PEB Private	r = − 0.125 *p* = 0.012*	r = − 0.090 *P* = 0.071	r = − 0.182 *p* < 0.001*	r = − 0.167 *p* = 0.001*	r = 0.181 *p* < 0.001*	–

MV: Material values; Env. Attitude: Environmental attitude; PEBs: Pro-environmental behaviors. Spearman’s correlation was used for associations.

*Denotes statistically significant difference at 0.05 level.

### Public pro-environmental behaviors

#### Public pro-environmental behaviors and their sociodemographic determinants (objectives 1 and 2)

Participation in public PEBs was generally low among students (Table 3). However, all public PEBs were significantly associated with at least one sociodemographic factor (*p* < 0.05). Nationality was significantly associated with group membership (*p* = 0.007) and donation behavior (*p* < 0.001), students from GCC and OAC reporting higher engagement than Egyptians. Gender was significantly associated with donation behavior, with males more likely to donate than females (*p* = 0.035). Age was significantly higher among those who donated to environmental groups (*p* = 0.001) or attended conferences on environmental issues (*p* = 0.002). Pocket money was significantly higher among students who donated (*p* < 0.001).

**Table 3:** Public pro-environmental behaviors by student characteristics (n = 405).

	Response	Group member		Gave money		Attended conference	
		n (%)		n (%)		n (%)	
All participants	No	390 (96.3%)		345 (85.2)		332 (82.0%)	
(n = 405)	Yes	15 (3.7%)		60 (14.8%)		73 (18.0%)	

		n (%)	*p*	n (%)	*p*	n (%)	p
Nationality							
Egyptian (n = 225)	No	220 (97.78%)	0.007*	208 (92.44%)	** < 0.001**	189 (84%)	0.214
	Yes	5 (2.22%)		17 (7.56%)		36 (16%)	
OAC (n = 140)	No	135 (96.43%)		106 (75.71%)		114 (81.43%)	
	Yes	5 (3.57%)		34 (24.29%)		26 (18.57%)	
GCC (n = 40)	No	35 (87.50%)		31 (77.50%)		29 (72.50%)	
	Yes	5 (12.50%)		9 (22.50%)		11 (27.50%)	
Gender							
Male	No	253 (96.56%)	0.698	216 (82.44%)	0.035*	209 (79.77%)	0.118
(n = 262)	Yes	9 (3.44%)		46 (17.56%)		53 (20.23%)	
Female	No	137 (95.80%)		129 (90.21%)		123 (86.01)	
(n = 143)	Yes	6 (4.20%)		14 (9.79%)		20 (13.99%)	

	Response	Group member		Gave money		Attended conference	
		Mean ± SD [95% CI for Mean] Median (IQR)	*p*	Mean ± SD [95% CI for Mean] Median (IQR)	*p*	Mean ± SD [95% CI for Mean] Median (IQR)	p
Age	No	22.45 ± 1.29	0.600	22.40 ± 1.31	0.001*	22.40 ± 1.33	0.002*
		[22.32–22.58]		[22.26–22.54]		[22.26–22.55]	
		22.00 (1.00)		22.00 (1.00)		22.00 (1.00)	
	Yes	22.60 ± 1.35		22.75 ± 1.11		22.68 ± 1.07	
		[21.85–23.35]		[22.46–23.04]		[22.43–22.94]	
		22.50 (2.00)		23.00 (1.00)		23.00 (1.00)	
Pocket money	No	9955.40 ± 14,834.90	0.107	9142.03 ± 13,963.77	< 0.001*	9998.75 ± 15,312.18	0.527
		[8424.19–11,486.60]		[7603.84–10,680.23]		[8276.29–11,721.22]	
		3000.00 (13,500.00)		3000.00 (11,500.00)		3250.00 (13,500.00)	
	Yes	27,200.00 ± 43,505.59		18,591.38 ± 27,110.29		13,168.87 ± 22,737.89	
		[2080.62–52,319.38]		[11463.09–25,719.67]		[7786.90–18,550.84]	
		13,000.00 (23,125.00)		14,650.00 (17,500.00)		4000.00 (17,500.00)	

IQR: Interquartile range; OAC: Other Arab countries; GCC: Gulf Cooperation Council. Mann–Whitney U was used for group comparisons.

*Denotes statistically significant difference at 0.05 level.

#### Interrelationships between material values, environmental attitudes, and public pro-environmental behaviors (objective 3)

MV and environmental attitudes were variably associated with public PEBs (Table 4). MV-success and MV-centrality were significantly associated with group membership and conference attendance respectively (*p* < 0.05). Giving money was not associated with MV or environmental attitudes.

**Table 4:** Association between public pro-environmental behaviors, material values, and environmental attitudes (n = 405).

	Response	Group member		Gave money		Attended conference	
		Mean ± SD [95% CI for Mean] Median (IQR)	*p*	Mean ± SD [95% CI for Mean] Median (IQR)	*p*	Mean ± SD [95% CI for Mean] Median (IQR)	p
MV success	No	2.23 ± 0.69	0.025*	2.25 ± 0.71	0.935	2.25 ± 0.72	0.961
		[2.16–2.30]		[2.17–2.32]		[2.17–2.33]	
		2.20 (1.00)		2.20 (1.00)		2.20 (1.00)	
	Yes	2.72 ± 0.94		2.26 ± 0.70		2.24 ± 0.65	
		[2.19–3.24]		[2.07–2.44]		[2.09–2.40]	
		2.70 (1.25)		2.20 (1.05)		2.20 (0.80)	
MV happiness	No	2.95 ± 0.75	0.800	2.96 ± 0.76	0.840	2.97 ± 0.77	0.225
		[2.88–3.03]		[2.87–3.04]		[2.89–3.05]	
		3.00 (1.00)		3.00 (1.00)		3.00 (1.05)	
	Yes	2.93 ± 0.79		2.94 ± 0.67		2.88 ± 0.64	
		[2.49–3.37]		[2.76–3.11]		[2.73–3.03]	
		2.90 (1.20)		3.00 (1.00)		2.80 (1.00)	
MV centrality	No	2.92 ± 0.64	0.443	2.94 ± 0.64	0.165	2.96 ± 0.64	0.006*
		[2.86–2.98]		[2.87–3.01]		[2.89–3.03]	
		3.00 (1.00)		3.00 (1.00)		3.00 (1.00)	
	Yes	3.08 ± 0.66		2.85 ± 0.66		2.75 ± 0.60	
		[2.71–3.44]		[2.67–3.02]		[2.61–2.89]	
		3.00 (1.25)		2.80 (1.00)		2.80 (0.80)	
Total MV	No	2.70 ± 0.53	0.155	2.72 ± 0.54	0.529	2.73 ± 0.55	0.094
		[2.65–2.76]		[2.66–2.77]		[2.67–2.79]	
		2.66 (0.73)		2.73 (0.80)		2.73 (0.75)	
	Yes	2.91 ± 0.66		2.68 ± 0.52		2.63 ± 0.46	
		[2.54–3.28]		[2.54–2.81]		[2.52–2.73]	
		2.90 (0.93)		2.66 (0.75)		2.60 (0.60)	
Env. attitude	No	4.41 ± 0.52	0.144	4.41 ± 0.49	0.448	4.41 ± 0.50	0.853
		[4.36–4.46]		[4.35–4.46]		[4.36–4.47]	
		4.57 (0.57)		4.42 (0.57)		4.57 (0.57)	
	Yes	4.05 ± 0.82		4.35 ± 0.74		4.34 ± 0.68	
		[3.60–4.51]		[4.16–4.54]		[4.18–4.49]	
		4.35 (1.32)		4.57 (0.71)		4.57 (0.71)	

IQR: Interquartile range; MV: Material values; Env. Attitude: Environmental Attitude. Mann–Whitney U was used for group comparisons.

*Denotes statistically significant difference at 0.05 level.

### Predictors of pro-environmental behaviors (objective 4)

Multivariate analyses explored predictors of PEBs (Table 5). Linear regression was used for private PEBs and revealed that nationality and environmental attitudes were significant predictors (*p* < 0.05). Logistic regression was used for public PEBs, and showed that nationality, and certain subscales of MV were significant predictors (*p* < 0.05).

**Table 5:** Multivariate analysis of private and public pro-environmental behaviors among medical students (n = 405).

	Linear regression		Logistic regression	
Factor	Private PEBs	Group member	Gave money	Attended conference
	(B [95% CI],* p*)	OR [95% CI], *p*	OR [95% CI], *p*	OR [95% CI], *p*
Nationality^$^				
(OAC)	0.368 [0.148–0.588], 0.001*	1.699 [0.376–7.679], 0.491	3.219 [1.512–6.814], 0.002*	0.863 [0.436–1.709], 0.672
(GCC)	0.184 [− 0.157–0.524], 0.289	4.896 [0.815–29.401], 0.082	2.791 [0.957–8.144], 0.060	1.296 [0.487–3.447], 0.603
Gender^%^	0.151 [− 0.037–0.340], 0.116	1.910 [0.571–6.395], 0.294	0.687 [0.339–1.393], 0.298	0.809 [0.440–1.487], 0.495
Age	0.015 [− 0.055–0.085], 0.668	1.080 [0.759–1.536], 0.668	1.049 [0.867–1.269], 0.624	1.139 [0.942–1.377]. 0.179
Pocket money	< 0.001 [0.000–0.000], 0.736	1.000 [1.000–1.000], 0.173	1.000 [1.000–1.000], 0.153	1.000 [1.000–1.000], 0.326
MV success	− 0.001 [− 0.144–0.141], 0.984	3.121 [1.159–8.404], 0.024*	1.280 [0.776–2.110], 0.333	1.273 [0.814–1.990], 0.290
MV happiness	0.007 [− 0.122–0.135], 0.919	0.761 [0.320–1.811], 0.537	0.989 [0.629–1.555], 0.961	0.883 [0.589–1.324], 0.548
MV centrality	− 0.200 [− 0.355–0.045], 0.011*	0.757 [0.276–2.073], 0.588	0.722 [0.422–1.234], 0.233	0.5844 [0.357–0.956], 0.032*
Env. Attitude	0.180 [0.018–0.342], 0.030*	0.730 [0.324–1.643], 0.447	0.938 [0.563–1.561], 0.805	0.826 [0.518–1.315], 0.826

Env. Attitude: Environmental attitude. B: Unstandardized Beta, CI: Confidence interval, OR: odds ratio, CI: Confidence interval, OAC: Other Arab countries, GCC: Gulf Cooperation Council, MV: Material values.

^$^: Reference category for nationality is Egyptian. %: Reference category for gender is male. Total MV was excluded from the regression due to multicollinearity with other predictors (tolerance < 0.001).

* Denotes statistically significant difference at 0.05 level.

**Fig. 1:**
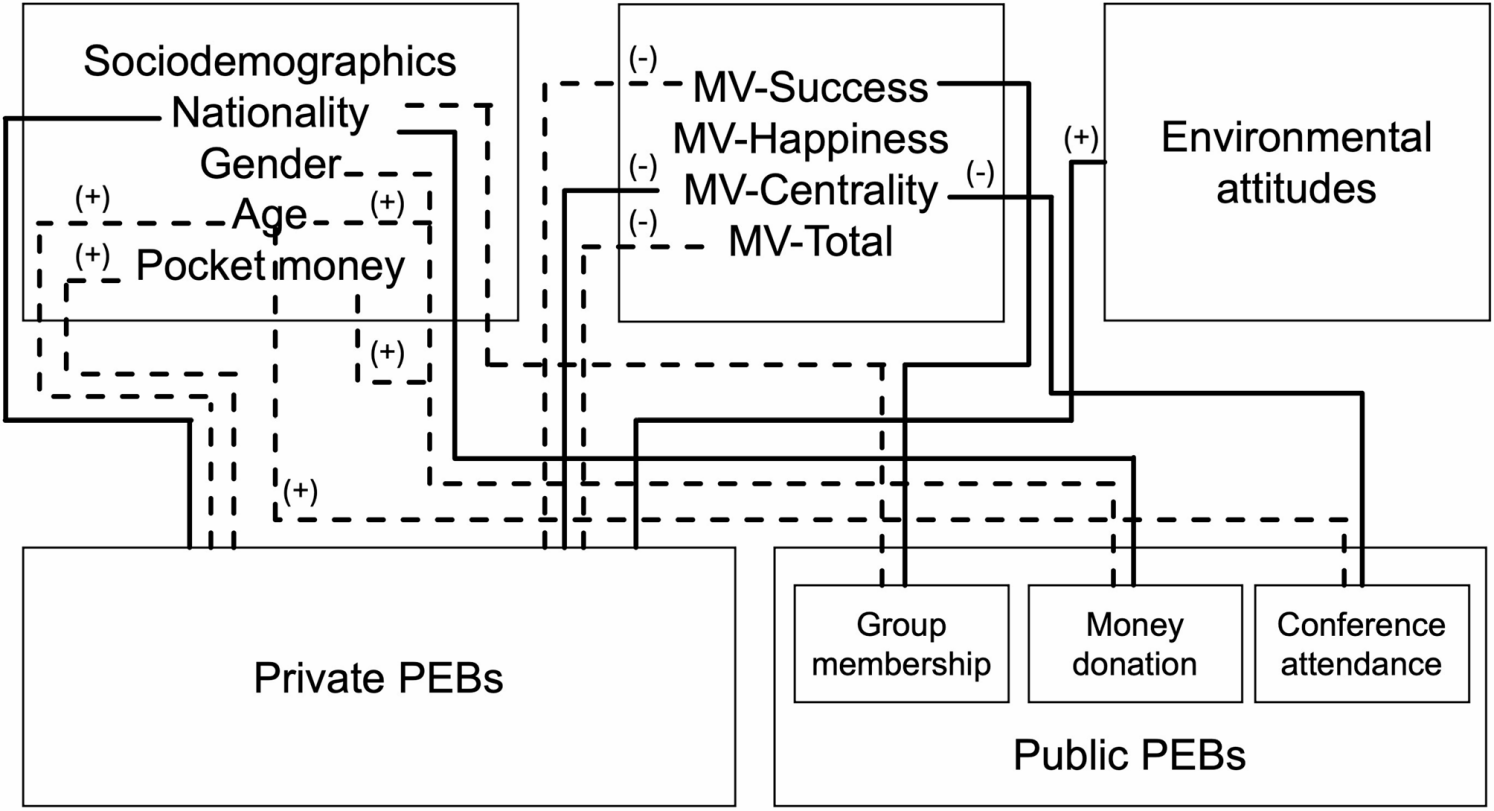
Final relationships for private and public pro-environmental behaviors. MV: Material values; PEBs: Pro-environmental behaviors. Lines detonate significant associations/correlations. Solid lines denote significance in regression. Dashed lines denote significance in bivariate analysis only. (+) denotes positive association/ correlation. (−) denotes negative association/ correlation.

Figure 1 shows the final findings of significant relationships of PEBs (private and public) and sociodemographics, MV and environmental attitude.

## Discussion

This study examined the intersection of MV, environmental attitudes, and PEBs among medical students in a coastal context. Overall, students demonstrated moderate MV and private PEBs, relatively high environmental attitudes, and low public PEBs. MV was inversely associated with environmental attitudes and private PEBs, while environmental attitudes were positively associated with private PEBs. In multivariate analyses, only a limited number of predictors—primarily MV dimensions and nationality—remained significant. These findings should be interpreted in light of the sampling approach, as quota sampling via online student groups may have introduced self-selection bias, potentially over-representing students with greater environmental interest.

Materialism among participants was moderate, aligning with prior studies highlighting the pervasive influence of consumerist norms among youth^8,17,18,30^. This intermediate level suggests that MV are neither negligible nor dominant, but remain relevant targets for educational strategies aimed at reinforcing non-materialistic and sustainability-oriented values.

Sociodemographic differences in MV were limited and largely attenuated after adjustment. Lower MV-happiness scores among GCC students are consistent with evidence linking stronger material aspirations to lower-income settings^31^. Age-related declines in MV, reported elsewhere^5,8,31^, were observed but should be interpreted cautiously given the narrow age range and the recruitment of participants from a single Academic cohort. Gender differences were minimal, apart from slightly higher MV-centrality among females, echoing associations with market-driven or appearance-related pressures^32,33^. While higher pocket money correlated with MV-centrality, consistent with lifestyle inflation dynamics^34^, total MV did not vary by nationality, likely due to averaging across heterogeneous subscales.

Environmental attitudes were generally high, similar to some regional findings^16,25^. Additionally, minimal sociodemographic variation was observed, consistent with other research findings^25,35,36^. This homogeneity may reflect shared professional socialization within medical education.

Private PEBs were moderate—consistent with prior work^8,18,28^—but lower than reports from other settings^18^, possibly reflecting differences in measurement, as behaviors were restricted to those performed explicitly for environmental reasons. Students from the OAC group, many of whom come from conflict-affected or resource-constrained settings, reported the highest private PEBs. These patterns likely reflect social and environmental norms shaped by necessity or strong community expectations, consistent with the theory of planned behavior (TPB)^37^. Financially advantaged students exhibited higher private PEBs, possibly due to greater access to eco-friendly products. This finding aligns with the concept of “princely” green behaviors, where environmentalism is partly tied to social signaling^8^.

Materialism demonstrated a consistent inverse relationship with both environmental attitudes and private PEBs, in line with existing literature^6,7,15–18^, particularly for MV-success and MV-centrality. This supports the interpretation that materialistic orientations may constrain intrinsically motivated environmental engagement. Although some studies suggest that status-oriented consumption can align with PEBs^8^, the present findings likely capture more intrinsic motivations due to the measurement approach.

In adjusted models, MV-centrality and nationality emerged as the primary predictors of private PEBs, whereas environmental attitudes were no longer significant. This pattern suggests that value orientations may exert stronger independent effects on behavior than attitudes alone, consistent with prior findings^16^.

Public PEBs were consistently low^27,35^, with distinct predictors compared to private behaviors. Group membership was predicted solely by MV-success, which may reflect the role of social norms and perceived expectations as described in the TPB^37^. Donating behavior and group participation were primarily shaped by nationality in adjusted models, indicating the influence of cultural and contextual factors. Attendance at environmental events was inversely associated with MV-centrality, consistent with the value–attitude–behavior framework^10^. Collectively, these findings reinforce the domain-specific nature of PEBs and their differing determinants.

These results highlight the importance of integrating sustainability into medical education, with emphasis on fostering intrinsic motivation and critical reflection on value orientations. Interventions should be context-sensitive to ensure inclusivity across socioeconomic and cultural backgrounds.

This study contributes to the literature by jointly examining MV, environmental attitudes, and both public and private PEBs within a cohort of future physicians, while accounting for cross-cultural variation among Arab medicall students. The distinction between public and private PEBs further clarifies that these behaviors are not uniform and should be addressed through tailored approaches.

Limitations include the cross-sectional design, which precludes causal inference, and the use of quota sampling via online platforms, introducing potential self-selection bias. The single-institution setting limits generalizability, and self-reported measures may be subject to social desirability bias. Additionally, modifications to the environmental attitudes scale and publlic PEBs may affect comparability with other studies.

## Conclusion

This study, conducted among medical students in a coastal city, demonstrated that MV were inversely associated with environmental attitudes and private PEBs, whereas public PEBs were primarily shaped by sociodemographic factors, with distinct predictors for environmental group membership, donations to environmental groups, and attendance of conferences on environmental issues. These findings support the view that PEBs are domain-specific and influenced by both value orientations and contextual factors, including cultural background and resource environments.

From a practical perspective, the findings highlight the importance of educational strategies that extend beyond knowledge dissemination to address underlying value orientations and support intrinsic motivation for environmental engagement. Integrating sustainability and climate-related content into medical curricula—alongside experiential learning and opportunities for civic participation—may help strengthen students’ engagement with pro-environmental practices. Tailoring such approaches to cultural and economic contexts may further enhance their relevance and inclusivity.

Further validation of culturally adapted environmental attitude scales is warranted. Future research should employ longitudinal or experimental designs to better understand how value orientations and environmental attitudes develop over time and how they relate to different forms of PEBs. Such work would provide a stronger evidence base for designing interventions to promote environmentally responsible practices among future healthcare professionals.

## Supplementary Information

Below is the link to the electronic supplementary material.

Supplementary Material 1

Supplementary Material 2

Supplementary Material 3

## Data Availability

The datasets generated during and/or analysed during the current study are available from the corresponding author on reasonable request.

## Abbreviations

GCC: Gulf Cooperation Council
MV: Material values
OAC: Other Arab countries
PEBs: Pro-environmental behaviors
TPB: Theory of planned behavior
