# Supplementary material for: Material values, environmental attitudes, and pro-environmental behaviors among future physicians in a coastal setting
Source: Sci Rep. 2026 Apr 23;16:13259. doi: 10.1038/s41598-026-47832-9 (PMC13106701; doi:10.1038/s41598-026-47832-9)
Supplement: Supplementary file 1 — Supplementary Material 1 [file 41598_2026_47832_MOESM1_ESM.pdf]

Supplementary file S1

Study tool

Material Values, Environmental Attitudes, and Pro-Environmental Behaviors among Future Physicians in a Coastal Setting

You are invited to participate in a research study aimed at assessing the material values, environmental attitudes, and pro-environmental behaviors of fifth-year medical students at Alexandria University. Your participation will help understand these values and attitudes and how they relate to pro-environmental behaviors.

The questionnaire is divided into four parts:
- Part 1: General characteristcs of the participants.
- Part 2: Material Values Scale
- Part 3: Environmental attitudes
- Part 4: Pro-environmental behaviors

Your participation is completely optional and voluntary. You have the right not to participate in the study, and you may withdraw at any time, without any negative consequences for you if you do not participate or withdraw. None of these questions trace back to your personal identity. Your information and data will remain completely confidential, and only researchers conducting the study will be able to access it.

- I agree to participate in the study, on a voluntary basis.
- I do not agree to participate in the study.

Part 1: General characteristics:

1. Age: ___ years.

2. Gender: ( ) Male.
( ) Female.

3. Nationality:

( ) Egyptian.	( ) Jordanian.	( ) Syrian
( ) Palestinian.	( ) Sudanese.	( ) Iraqi.
( ) Yemeni.	( ) Libyan.	( ) Kuwaiti.
( ) Omani.	( ) Bahraini.	( ) Saudi Arabian.

4. Monthly pocket money: _______ EGP.

Part 2: Material Values Scale:

Below is a series of statements concerning material values. Please work quickly and record your first impression by indicating the degree to which you agree or disagree with the statement, where 1= Strongly disagree, and 5= Strongly agree.

	1= Strongly disagree	2= Disagree	3= Uncertain	4= Agree	5= Strongly agree
1. I like to own things that impress people.	1	2	3	4	5
2. I admire people who own expensive homes, cars, and clothes.	1	2	3	4	5
3. The things I own say a lot about how well I'm doing in life.	1	2	3	4	5
4. Some of the most important achievements in life include acquiring material possessions.	1	2	3	4	5
5. I don't place much emphasis on the amount of material objects people own as a sign of success.	1	2	3	4	5
6. I usually buy only the things I need.	1	2	3	4	5
7. I like a lot of luxury in my life.	1	2	3	4	5
8. I try to keep my life simple, as far as possessions are concerned.	1	2	3	4	5
9. Buying things gives me a lot of pleasure.	1	2	3	4	5
10. The things I own aren't all that important to me.	1	2	3	4	5
11. I'd be happier if I could afford to buy more things.	1	2	3	4	5
12. My life would be better if I owned certain things Idon't have.	1	2	3	4	5
13. I have all the things I really need to enjoy life.	1	2	3	4	5
14. It sometimes bothers me quite a bit that I can't afford to buy all the things I'd like.	1	2	3	4	5
15. I wouldn't be any happier if I owned nicer things.	1	2	3	4	5

Part 3: Environmental attitudes (adapted version)

Below is a series of statements concerning environmental attitudes. There are no right or wrong answers. Please work quickly and record your first impression by indicating the degree to which you agree or disagree with the statement, where 1= Strongly disagree, and 5= Strongly agree.

	1= Strongly disagree	2= Disagree	3= Uncertain	4= Agree	5= Strongly agree
1. A clean environment is a shared right for all individuals.	1	2	3	4	5
2. I am prepared to give up certain conveniences to help solve environmental problems.	1	2	3	4	5
3. Prioritizing environmental protection hinders industrial progress.	1	2	3	4	5
4. All living organisms have an equal right to exist.	1	2	3	4	5
5. Humans have a greater negative impact on the environment than other living beings.	1	2	3	4	5
6. Environmental education should begin at an early stage in life.	1	2	3	4	5
7. Humans are justified in harming nature to meet their survival needs.	1	2	3	4	5

Part 4: Pro-environmental behaviors (adapted version)

A. Below is a list of behaviors (in your private sphere) that affect the environment. Rate each one based on how often you perform it, where 1= Never, and 5= Always.

	1= Never	2= Rarely	3= Sometimes	4= Usually	5= Always
1. How often do you sort recyclable materials (e.g., glass, plastic, paper)?	1	2	3	4	5
2. How often do you purchase produce grown without pesticides?	1	2	3	4	5
3. How often do you reduce car use (riding/ driving) for environmental reasons?	1	2	3	4	5
4. How often do you limit household energy or fuel consumption for environmental reasons?	1	2	3	4	5
5. How often do you conserve or reuse water for environmental reasons?	1	2	3	4	5
6. How often do you avoid purchasing products due to environmental concerns?	1	2	3	4	5

B. Below is a list of questions on behaviors (in the public sphere) that affect the environment. Rate each one according to whether you did it or not, where 0= you did not do it, and 1= you did it.

	0= No	1= Yes
1. Are you currently a member of an environmental organization?	0	1
2. Have you donated to an environmental organization in the past five years?	0	1
3. Have you attended an event or meeting on environmental issues in the past five years?	0	1

Thank you for your time.
