# Supplementary material for: Material values, environmental attitudes, and pro-environmental behaviors among future physicians in a coastal setting
Source: Sci Rep. 2026 Apr 23;16:13259. doi: 10.1038/s41598-026-47832-9 (PMC13106701; doi:10.1038/s41598-026-47832-9)
Supplement: Supplementary file 2 — Supplementary Material 2 [file 41598_2026_47832_MOESM2_ESM.pdf]

Supplementary file S2

Adapted full environmental attitude scale items

Scale item	Relevant sub-domain: Attitude towards environmental
1. A clean environment is a shared right for all individuals.	Solution
2. Most human activities negatively affect the environment.	Solution
3. Modern lifestyles are generally harmless to the environment. *(R)*	Solution
4. Economic growth does not necessarily lead to environmental harm. *(R)*	Problem
5. Environmental issues are confined to local areas rather than global concerns. *(R)*	Solution
6. I believe environmental problems can be effectively addressed in the near future.	Solution
7. Environmental issues are not my personal responsibility. *(R)*	Solution
8. I am prepared to give up certain conveniences to help solve environmental problems.	Solution
9. Prioritizing environmental protection hinders industrial progress.	Solution
10. Advances in technology often contribute to environmental damage.	Problem
11. All living organisms have an equal right to exist.	Solution
12. Humans have a greater negative impact on the environment than other living beings.	Problem
13. The balance of natural ecosystems is fragile and easily disrupted.	Problem
14. Environmental education should begin at an early stage in life.	Solution
15. Humans are justified in harming nature to meet their survival needs. *(R)*	Problem

(R): denotes reverse coded items

Final 7-items adapted environmental attitude scale

Scale item
1. A clean environment is a shared right for all individuals.
2. I am prepared to give up certain conveniences to help solve environmental problems.
3. Prioritizing environmental protection hinders industrial progress.
4. All living organisms have an equal right to exist.
5. Humans have a greater negative impact on the environment than other living beings.
6. Environmental education should begin at an early stage in life.
7. Humans are justified in harming nature to meet their survival needs. *(R)*

(R): denotes reverse coded items
