# Supplementary material for: Material values, environmental attitudes, and pro-environmental behaviors among future physicians in a coastal setting
Source: Sci Rep. 2026 Apr 23;16:13259. doi: 10.1038/s41598-026-47832-9 (PMC13106701; doi:10.1038/s41598-026-47832-9)
Supplement: Supplementary file 3 — Supplementary Material 3 [file 41598_2026_47832_MOESM3_ESM.pdf]

Supplementary file S3

Parametric tests of significance for bivariate analysis of material values, environmental attitudes and pro-environmental behaviors with sociodemographics.

Variable	MV success	MV happiness	MV centrality	Total MV	Total attitude	Private PEBs
Nationality	p= 0.448	**p= 0.007***	p= 0.447	p= 0.200	p= 0.266	**p= 0.001***
Gender	p= 0.427	p= 0.169	**p< 0.001***	p= 0.685	**p= 0.019***	p= 0.665
Age	r= -0.120	r= -0.004	r= -0.131	r= -0.106	r= -0.037	r= 0.098
	**p= 0.016***	p= 0.932	**p= 0.008***	**p= 0.033***	p= 0.460	**p= 0.049***
Pocket money	r= 0.009	r= -0.039	r= 0.089	r= 0.021	r= -0.090	r= 0.083
	p= 0.860	p= 0.445	p= 0.085	p= 0.683	p= 0.082	p= 0.106

MV: Material values; Env. Attitude: Environmental attitude; PEBs: Pro-environmental behaviors.
Parametric tests (independent t-test, one-way ANOVA, and Pearson's correlation) were conducted.
* Denotes statistically significant difference at 0.05 level.
Grey shaded cells denote associations that were significant and became insignificant, or vice versa, after applying parametric analysis.

Parametric correlations for study variables.

	MV Success	MV Happiness	MV Centrality	Total MV	Total Attitude	PEBs Private
MV Success	-	-	-	-	-	-
MV Happiness	-	-	-	-	-	-
MV Centrality	-	-	-	-	-	-
Total MV	-	-	-	-	-	-
Total attitude	r= -0.220 **p= <0.001***	r= -0.070 p= 0.159	r= -0.052 p= 0.296	r=-0.149 **p= 0.003***	-	-
PEB Private	r= -0.113 **p= 0.023***	r= -0.066 P= 0.182	r= -0.136 **p= 0.006***	r= -0.1344 **p= 0.007***	r= 0.122 **p= 0.014***	-

MV: Material values; Env. Attitude: Environmental attitude; PEBs: Pro-environmental behaviors.
Pearson's correlation was conducted.
* Denotes statistically significant difference at 0.05 level.
Significance/ insignificance status remained similar to non-parametric analysis.

Parametric tests of significance for bivariate analysis of public pro-environmental behaviors with sociodemographics.

	Group member	Gave money	Attended conference
**Age**	p= 0.658	p= 0.055	p= 0.093
**Pocket money**	p= 0.163	**p= 0.012***	p= 0.267

Independent t-test was conducted.

* Denotes statistically significant difference at 0.05 level.

Grey shaded cells denote associations that were significant and became insignificant, after applying parametric analysis.

Parametric tests of significance for bivariate analysis of public pro-environmental behaviors with material values and environmental attitudes.

	Group member	Gave money	Attended conference
**MV success**	**p= 0.010***	p= 0.951	p= 0.913
**MV happiness**	p= 0.897	p= 0.841	p= 0.361
**MV centrality**	p= 0.364	p= 0.291	**p= 0.012***
**Total MV**	p= 0.155	p= 0.629	p= 0.143
**Env. Attitude**	p= 0.115	p= 0.593	p= 0.370

MV: Material values; Env. Attitude: Environmental attitude

Independent t-test was conducted.

* Denotes statistically significant difference at 0.05 level.

Significance/ insignificance status remained similar to non-parametric analysis.
